# Comparison of Supreme laryngeal mask airway versus endotracheal intubation for airway management during general anesthesia for cesarean section: a randomized controlled trial

**DOI:** 10.1186/s12871-019-0792-9

**Published:** 2019-07-08

**Authors:** Wei Yu Yao, Shi Yang Li, Yong Jin Yuan, Hon Sen Tan, Nian-Lin R. Han, Rehena Sultana, Pryseley N. Assam, Alex Tiong-Heng Sia, Ban Leong Sng

**Affiliations:** 1 Department of Anesthesiology and Perioperative Medicine, Quanzhou Macare Women’s Hospital, Quanzhou, Fujian Province China; 2 Department of Anesthesiology, Qinghai University Affiliated Hospital, Xining, Qinghai Province China; 3 Department of Women’s Anaesthesia, KK Women’s and Children’s Hospital, 100 Bukit Timah Road, Singapore, Singapore; 4 Division of Clinical Support Services, KK Women’s and Children’s Hospital, 100 Bukit Timah Road, Singapore, Singapore; 5 Center for Quantitative Medicine, Duke-NUS Medical School, 8 College Road, Singapore, Singapore; 6 Singapore Clinical Research Institute, 31 Biopolis Way, Singapore, Singapore; 7 Anaesthesiology and Perioperative Sciences Academic Clinical Program, Duke-NUS Medical School, 8 College Road, Singapore, Singapore

**Keywords:** Laryngeal mask airway, Obstetric, Cesarean section, General anesthesia

## Abstract

**Background:**

The obstetric airway is a significant cause of maternal morbidity and mortality. Endotracheal intubation is considered the standard of care but the laryngeal mask airway (LMA) has gained acceptance as a rescue airway and has been incorporated into the obstetric airway management guidelines. In this randomized controlled equivalence trial, we compared the Supreme LMA (SLMA) with endotracheal intubation (ETT) in managing the obstetric airway during cesarean section.

**Methods:**

Parturients who underwent elective cesarean section under general anesthesia were randomized to receive either an SLMA or ETT as their airway device. Our primary outcome was first-attempt insertion success. Successful insertion was defined as adequate bilateral air entry with auscultation and the presence of end-tidal carbon dioxide on the capnogram. The first-attempt insertion success rate was compared using the Chi-Square test. Secondary outcomes included time-to-ventilation, seal pressure, ventilation/hemodynamic parameters, occurrence of clinical aspiration, fetal outcomes, and maternal side effects associated with the airway device.

**Results:**

We recruited 920 parturients (460 SLMA, 460 ETT) who underwent elective cesarean section under general anesthesia. Patient characteristics were similar between the groups. First attempt success was similar (Odds Ratio--OR_SLMA/ETT_: 1.00 (95%CI: 0.25, 4.02), *p* = 1.0000). SLMA was associated with reduced time to effective ventilation (Mean Difference--MD -22.96; 95%CI: − 23.71, − 22.21 s) compared to ETT group (*p* <  0.0001). Ventilation parameters, maternal and fetal outcomes were similar between the groups, and there was no aspiration.

**Conclusions:**

SLMA could be an alternative airway management technique for a carefully selected low-risk obstetric population, with similar insertion success rates, reduced time to ventilation and less hemodynamic changes compared with ETT. Our findings are consistent with the airway guidelines in recommending the second-line use of LMA in the management of the obstetric airway.

**Trial registration:**

The study was registered at http://www.clinicaltrials.gov, identifier: NCT01858467, retrospectively registered. Date of registration: May 21, 2013.

## Background

Airway complications are one of the main causes of anesthetic-related obstetric adverse events in developed countries, with the incidence of failed intubation estimated to be around 0.4% [1–5]. Obstetric airway management is complex, due to the unique challenges posed by the physiological changes of pregnancy, including reduced oncotic pressure, oxytocin-related water retention, and Valsalva maneuver during labor leading to mucosal capillary engorgement and edema. This is often confounded by inadequate fasting time, urgency of cesarean section requiring general anesthesia, and the need to balance the potential conflicting needs of the mother and fetus [6–8].

The introduction of second-generation supraglottic airway devices (SAD) such as the Proseal Laryngeal Mask Airway (LMA) and Supreme LMA (SLMA) with a double-lumen specifically designed to physically separate the respiratory and alimentary tract, reduces the risk of aspiration [8–12] and has changed obstetric airway management. The first Difficult Airway Society (DAS) guideline for managing an unanticipated difficult intubation published in 2004 incorporated the LMA as a second-line rescue device, though obstetric patients were specifically excluded [13]. Subsequently, the important role of the LMA in obstetric difficult airway management was recognized in the first obstetric-specific guideline jointly released by Obstetric Anaesthetists’ Association (OAA) and DAS [14]. In addition, the OAA-DAS guideline recommends an active decision process that takes into consideration the maternal risk of proceeding without endotracheal intubation versus the fetal risk of delayed surgery. The guideline also drew attention to the traditional notion that general anesthesia for cesarean section is unacceptable without endotracheal intubation, due to concerns of increased risk of pulmonary aspiration.

The practice of rapid sequence induction (RSI) and ETT as the standard of care for obstetric airway management originated from concerns of pulmonary aspiration of gastric contents following general anesthesia [15]. Current obstetric anesthesia practice is associated with a low but significant incidence of pulmonary aspiration of about 0.1% [3]. The increasing use of neuraxial anesthesia, aspiration prophylaxis, stricter adherence to fasting guidelines, and airway control via RSI and ETT for general anesthesia are factors that may enhance the margin of safety in this respect. However, rising rates of obesity and other risk factors for pulmonary aspiration may necessitate endotracheal intubation as standard of care.

Recently, the efficacy and safety of LMA use in selected patients undergoing cesarean section under general anesthesia has been demonstrated in several prospective and retrospective cohort studies [12, 16–18]. However, to date, there are no randomized controlled trials comparing airway outcomes of the LMA compared to ETT in cesarean section. This prospective, randomized controlled trial aims to evaluate the first insertion attempt success rate of the SLMA compared to ETT in elective cesarean section under general anesthesia.

## Methods

This study was approved by the Institutional Review Board (approval obtained on 25th October 2012) at the Quanzhou Women’s and Children’s Hospital, Fujian Province, China, and registered at http://www.clinicaltrials.gov (NCT01858467). At the time of the study, the majority of cesarean sections at Quanzhou Women’s and Children’s Hospital were performed under general anesthesia largely due to patients’ request, with routine airway management using the SLMA in both elective and urgent cases [18]. The hospital cesarean section rate is 35% and the SLMA technique for general anesthesia is used in about 2000 deliveries annually, with endotracheal intubation employed as an alternative airway management.

We recruited parturients with singleton pregnancies aged 18 to 50 years old who are healthy or with well-controlled medical conditions (American Society of Anesthesiology; ASA 2) undergoing elective cesarean section under general anesthesia at Quanzhou Women’s and Children’s Hospital, China between May 2013 and July 2014. Written informed consent was obtained from every participant. Our exclusion criteria included parturients with body mass index (BMI) ≥ 35 kg/m^2^, anticipated difficult airway (modified Mallampati grade 4, or known upper respiratory tract or neck pathology) or self-reported gastro-esophageal reflux disease. All parturients were fasted for a minimum of 6 h. Once enrolled in the study, the parturients were randomized into 2 groups and allocation concealed using sealed opaque envelopes prepared by a statistician not involved in the study recruitment. The 2 groups were (1) SLMA group; and (2) ETT group. The parturients and independent assessors were blinded to the assigned group, but the investigators managing the airway were not blinded.

The induction of anesthesia and airway device insertion reflects the clinical standard at our study center [18]. As per our institutional standard, all parturients received premedication with intravenous ranitidine, following which electrocardiography, pulse oximetry, capnography and non-invasive blood pressure measurements were started. Following preoxygenation for three minutes, RSI was performed with cricoid pressure applied by a trained anesthetic nurse. Anesthesia was induced with intravenous propofol (2-3 mg/kg) and 100 mg succinylcholine. After the airway device was inserted, rocuronium (0.5 mg/kg) was used to maintain muscle relaxation and fentanyl (100mcg) was administered for intraoperative analgesia after the fetus was delivered. All cases regardless of assigned study groups were induced in similar fashion.

The SLMA size was chosen based on manufacturer’s recommendations, however, a more appropriate size could be selected based on parturient’s weight, BMI, and mouth opening at the discretion of the investigators. ETTs of intraluminal diameters between 6.5 to 7.0 mm were used, chosen at the investigators’ discretion. Three investigators (Yao, Li, and Yuan), each with more than five years of experience in the use of both SLMA and ETT as well as difficult airway management in general anesthesia, managed the airway for all parturients enrolled in this study.

In the SLMA group, SLMA insertion using the recommended single-handed rotational technique was performed with the maintenance of cricoid pressure. Subsequently, a manometer was used to inflate the cuff to 60 cmH_2_O, and we recorded the volume of air required to achieve this pressure. Next, the ability to ventilate was confirmed as evidenced by the presence of auscultation of bilateral air entry and carbon dioxide trace on capnography, after which cricoid pressure was released. Following successful SLMA placement, a pre-mounted #14 gastric tube was advanced through the gastric drainage channel, and confirmed adequate placement by: (1) aspiration of gastric fluid; or (2) injection of air into the orogastric tube and auscultating a “swoosh” over the epigastrium. We recorded the number of attempts needed for successful placement, and suctioning of the orogastric tube was performed before commencement of the surgery. Finally, SLMA seal pressure was determined by recording the peak airway pressure after closing the adjustable pressure-limiting valve while maintaining a fresh gas flowrate of 3 L/min into the closed circuit.

If required, the use of additional maneuvers such as chin lift, head extension, jaw thrust, or SLMA repositioning were permitted to maintain a patent airway. Failure to achieve successful SLMA placement (1) after 2 attempts, (2) within one minute, or (3) before desaturation occurred (oxygen saturation < 92%), would result in securing of the airway via direct laryngoscopy and endotracheal intubation. Cesarean section would commence if the following were met: (1) presence of a square-wave capnogram; (2) SLMA cuff pressure of 60cmH_2_O; (3) SLMA bite block positioned between the incisors; (4) successful orogastric tube placement and suctioning performed; and (5) recorded SLMA seal pressure was ≥20cmH_2_O.

In the ETT group, oral endotracheal intubation via direct laryngoscopy was performed using a Macintosh blade (size 3–4 based on anesthesiologist’s preference). The ETT cuff was inflated to 25 cmH_2_O using a manometer, and we recorded the volume of air needed to achieve this pressure. Following confirmation of the ability to ventilate, as evidenced by the auscultation of bilateral air entry and carbon dioxide trace on capnography, cricoid pressure was released. A #14 orogastric tube was then inserted, and the position confirmed, as above.

For all parturients irrespective of assigned group, successful placement of the airway device was confirmed by auscultation of bilateral breath sounds and the capnographic presence of end-tidal carbon dioxide. We defined an insertion attempt as the insertion and complete removal of the airway device, and recorded the number of attempts required. We also measured the time to effective airway placement, which we defined as the duration from when the device was picked up till the first carbon dioxide waveform was seen.

Intraoperative anesthesia management reflects the clinical practice at the study center [18]. Maintenance of general anesthesia was achieved with 1.5 to 2.0% sevoflurane in a 50% mix of nitrous oxide and oxygen, with all parturients positioned in left lateral tilt using a wedge. During anesthesia, complications including the loss of airway, inadequate oxygenation or ventilation, and bleeding into the airway device were recorded. Parturients were ventilated with a tidal volume of 6 to 10 mL/kg at 10 to 16 breaths/min, targeting an end-tidal carbon dioxide concentration of 30 to 40 mmHg. The presence of clinical signs of aspiration, including perioperative hypoxemia, lung auscultation revealing wheezing or crepitations, and postoperative dyspnea would trigger investigation of the parturient with bronchoscopy or chest X-rays.

The obstetricians were advised to reduce fundal pressure during delivery regardless of the device used. Upon completion of surgery, reversal of muscle paralysis followed by suctioning and removal of the orogastric tube were performed. After achieving adequate spontaneous respiration and consciousness (defined as when parturient was able to follow instructions to open her eyes and mouth), the airway devices were removed and inspected for blood. An independent assessor recorded the incidence of sore throat and hoarseness before discharge from the post-anesthesia care unit.

Our primary outcome was the first-insertion attempt success rate of the airway devices. Secondary anesthetic outcomes included:

1. time to effective ventilation; airway device seal pressure;
2. ventilation parameters (tidal volume, respiratory rate, peak ventilation pressure) to maintain effective oxygenation and ventilation, which we defined as SpO_2_ ≥ 92% and end-tidal carbon dioxide concentration of < 50 mmHg, achieved via inspired oxygen concentration ≤ 0.5, respiratory rate of 10 to 16 breaths/min, and tidal volume of 6 to 10 mL/kg; and
3. hemodynamic parameters including heart rate and blood pressure for 6 min after induction.

We also recorded the amount and pH of gastric aspirate; as well as the incidence of regurgitation (presence of clear/bile-stained fluid during surgery or removal of the airway device); incidence of aspiration (bile-stained fluid noted in the lung via bronchoscopy or suggestive radiographical signs), and the pH of SLMA laryngeal surface and ETT cuff. Apart from neonatal weight, the obstetric outcomes recorded included neonatal Apgar scores at 1 and 5 min and umbilical cord venous pH. Maternal satisfaction (question asked, “how would you rate your overall satisfaction with your anesthetic care from 0 to 100%”) was assessed one day after surgery by an independent assessor.

### Statistical analysis

The primary outcome measure of first-attempt success rate of SLMA insertion and secondary outcomes, namely incidence of regurgitation, aspiration, blood staining of the airway devices and voice hoarseness were treated as categorical data, with categories “*yes*” or “*no*”. All demographic, anesthetic and clinical categorical variables were summarized as frequency with the corresponding proportion; and continuous variables were expressed as mean (standard deviation (SD)) or median [interquartile range (IQR)], whichever applicable with respect to two study groups SLMA and ETT. Difference in outcomes between ETT and SLMA were represented as odds ratio (OR) and mean difference or median difference, whichever applicable, with 95% confidence interval for categorical and continuous data respectively. Difference in categorical and continuous variables between SLMA and ETT were tested using Chi-Square test and Student’s t-test or Mann-Whitney U test, whichever applicable, respectively. Significance level was set at *p*-value < 0.05 and all tests were two-sided. Data analysis was generated using SAS 9.3 software (SAS Institute Inc., Cary, NC, USA).

We performed a sample size calculation based on our previous experience estimate of 98% in first attempt insertion success rate of SLMA insertion [18]. An equivalence boundary of 0.034 i.e. (96.3, 99.7%) as the maximum difference between maximum likelihood estimates of the true proportions in the first attempt insertion success rate in SLMA and ETT use was considered as clinical equivalence. A sample size of 916 parturients (458 per group) would achieve 80% power with the above-mentioned equivalence limits, based on a one-sided test with a significance level of 2.5% and retrospective estimate in the first attempt success rate of 98%. We aimed to recruit a total of 920 parturients in this study.

## Results

**Fig. 1:**
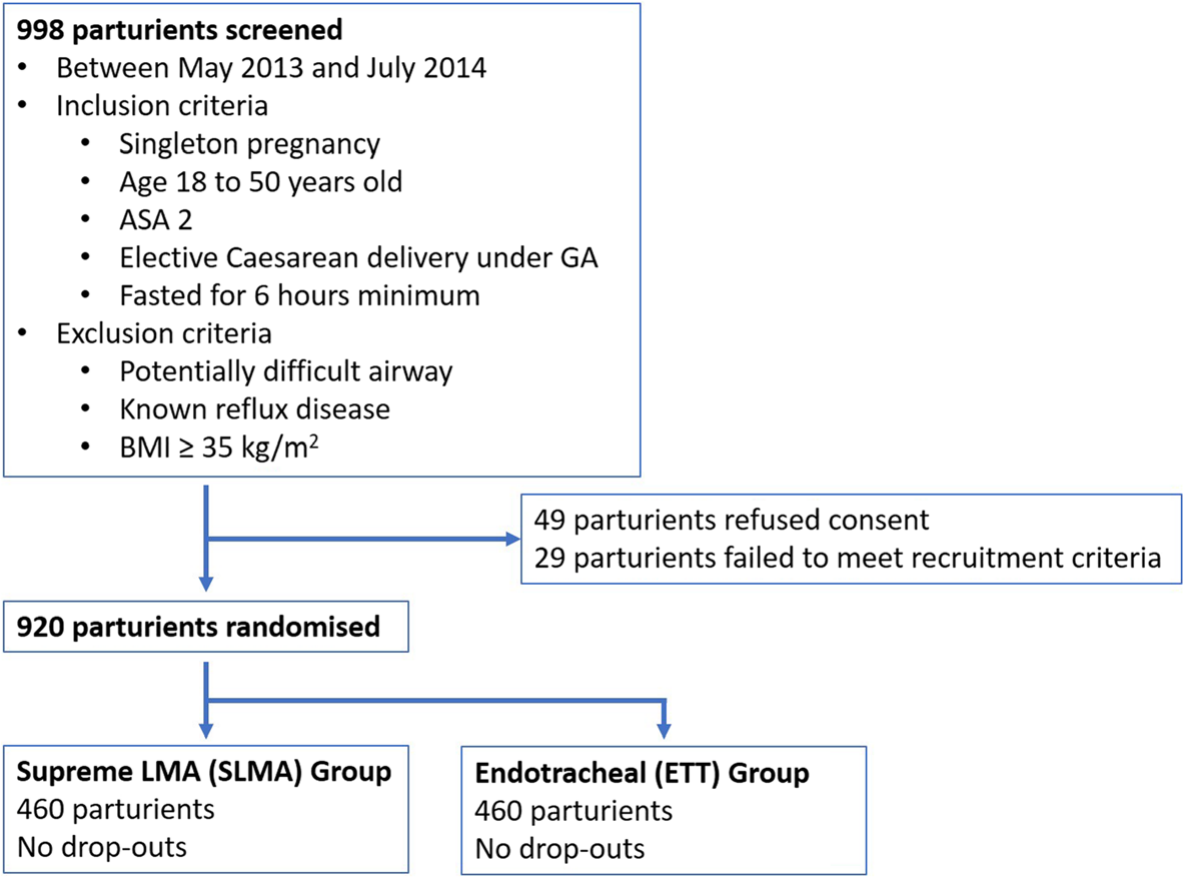
Consort diagram. 998 parturients who were planned for elective cesarean section in Quanzhou Women’s and Children’s Hospital were screened between May 2013 and July 2014. Inclusion criteria: singleton pregnancy, aged 18 to 50 years old, ASA 2, and fasted for at least 6 h. Parturients with potentially difficult airway, known reflux disease, or with BMI ≥ 35 kg/m^2^ were excluded. 49 parturients refused consent, and 29 did not meet the recruitment criteria. The remaining 920 parturients were randomized by opaque envelope to obtain 460 in each group. There were no dropouts or withdrawal

We screened 998 parturients between May 2013 and July 2014. There were 49 parturients who did not give consent and 29 parturients did not meet the recruitment criteria. A total of 920 parturients were randomized (460 assigned to each group) and there was no withdrawal or dropout (Fig. 1 consort diagram).

We summarized the demographic and clinical characteristics of the parturients in Table 1. There were no significant differences in maternal age, weight, height, ASA scores, Mallampati scores and fetal gestational age. There was no significant difference in duration from the time of anesthesia to delivery, and the overall duration of surgery.

**Table 1:** Baseline patient demographics and clinical characteristics of participants receiving SLMA or ETT while undergoing cesarean section under general anesthesia

Characteristics	SLMA (*n* = 460)	ETT (*n* = 460)	*p*-value
Age (years), mean (SD)	28.4 (4.1)	28.4 (4.0)	0.9869
Weight (kg), mean (SD)	65.6 (6.5)	65.0 (6.5)	0.1230
Height (cm), mean (SD)	158.9 (5.0)	158.1 (4.7)	**0.0130**
BMI (kg/m^2^), mean (SD)	26.0 (2.27)	26.0 (2.33)	0.9875
Mallampati, n (%)			0.8644
Mallampati 1	183 (39.8)	177 (38.5)	
Mallampati 2	244 (53.0)	252 (54.8)	
Mallampati 3	33 (7.2)	31 (6.7)	
Gestational age (weeks), mean (SD)	38.1 (1.2)	38.3 (1.2)	0.7422
Duration from anesthesia to delivery (min), mean (SD)	12.0 (7.9)	12.5 (7.9)	0.3307
Duration of surgery (min), mean (SD)	33.1 (10.5)	32.1 (10.1)	0.1524

Categorical values are compared using Chi-Square test and continuous variables are compared using two-sample Student’s t-test. *P*-values of 0.05 or less are considered significant

The anesthesia outcomes are presented in Table 2. The primary outcome first attempt success was similar in in both groups: SLMA 456/460 (99.1%), ETT 456/460 (99.1%), with OR of 1.00 (95%CI: 0.25, 4.02; *p* = 1.0000) when SLMA was compared with respect to ETT. We found there was a statistically significant reduction in time to effective ventilation (defined as the duration from when the device was picked up to appearance of first end-tidal carbon dioxide waveform) with MD of − 22.96 (95%CI: − 23.71, − 22.21; *p* <  0.0001) in SLMA insertion compared to ETT. There was a significant difference in seal pressure obtained (MD: -0.77; 95%CI: − 1.25, − 0.30; *p* = 0.0014). The rest of the ventilation parameters were similar between the groups, in terms of lowest tidal volume, lowest respiratory rate, highest peak airway pressure, seal minus highest peak airway pressure and lowest SpO_2_ achieved. We did not observe hypoxemia, laryngospasm or bronchospasm intra-operatively. Although the baseline heart rate and systolic blood pressure were similar, we found that SLMA insertion was associated with less hemodynamic alterations compared to endotracheal intubation, with lower heart rate and systolic blood pressure during the airway insertion period.

**Table 2:** Anesthetic outcomes of participants receiving either SLMA or ETT while undergoing cesarean section under general anesthesia

Characteristics	SLMA (*n* = 460)	ETT (*n* = 460)	Mean Difference / Odds ratio (95%CI)	*p*-value
Number of insertion attempts, n (%)				
First attempt	456 (99.1)	456 (99.1)	Reference	–
Second attempt or more	4 (0.9)	4 (0.9)	1.00 (0.25, 4.02)	1.0000
Time to effective ventilation (s), mean (SD)	16.1 (3.9)	39.1 (7.2)	-22.96 (−23.71, −22.21)	**< 0.0001**
Seal pressure (cmH_2_O), mean (SD)	27.1 (3.8)	27.9 (3.6)	-0.77 (−1.25, −0.30)	**0.0014**
Lowest tidal volume (ml), mean (SD)	430.9 (39.2)	429.2 (39.3)	1.76 (−3.32, 6.84)	0.4965
Lowest respiratory rate (breaths /min), median (IQR)	12 (12.0, 12.0)	12 (12.0, 12.0)	0.00 (0.00, 0.00)	0.7957
Highest peak airway pressure (cmH_2_O), mean (SD)	16.8 (3.4)	17.0 (3.6)	−0.14 (−0.59, 0.31)	0.5320
Seal pressure minus highest peak airway pressure (cmH_2_O), mean (SD)	10.3 (4.2)	10.9 (4.3)	−0.63 (−1.18, − 0.08)	**0.0250**
Lowest SpO2 (%), median (IQR)	99.0 (97.0, 99.0)	98.5 (97.0, 99.0)	0.50 (0.00, 0.00)	0.2109
Baseline systolic blood pressure (mmHg), mean (SD)	116.8 (8.6)	116.8 (11.2)	−0.002 (−1.297, 1.292)	0.9974
Systolic blood pressure 2 min after induction (mmHg), mean (SD)	114.0 (11.1)	133.9 (18.2)	−19.96 (−21.91, − 18.01)	**< 0.0001**
Systolic blood pressure 5 min after induction (mmHg), mean (SD)	103.7 (10.4)	111.2 (13.3)	−7.51 (−9.06, −5.97)	**< 0.0001**
Baseline heart rate (beats /min), mean (SD)	84.4 (10.3)	85.5 (10.1)	−1.04 (−2.36, 0.28)	0.1210
Heart rate 2 min after induction (beats /min), mean (SD)	93.6 (12.9)	105.4 (11.7)	−11.75 (− 13.34, − 10.16)	**< 0.0001**
Heart rate 5 min after induction (beats /min), mean (SD)	88.0 (13.3)	92.9 (11.9)	−4.87 (−6.5, −3.24)	**< 0.0001**

Difference between the two categories are expressed as mean difference or median difference and odds ratio with 95% confidence interval for continuous and categorical values, respectively. Categorical values are compared using Chi-Square test and continuous variables are compared using two-sample Student’s t-test or Wilcoxon test. *P*-values of 0.05 or less are considered significant

Maternal and fetal outcomes are summarized in Table 3. There were no significant mean differences in fetal weight, or median differences in APGAR scores at 1 and 5 min, and umbilical venous cord pH. The volume of gastric aspirate was similar between the groups, but the SLMA group had a lower pH with MD of − 0.06 (95%CI: − 0.11, − 0.02) compared to the ETT group (*p* = 0.0309). More importantly, the pH of the SLMA laryngeal surface was similar to that of the ETT cuff, suggesting the absence of gastric contents in the airway (− 0.001; 95%CI: − 0.004, 0.002 for both ETT and SLMA groups, *p* = 0.5613). There was no clinical suggestion of pulmonary aspiration.

**Table 3:** Fetal and maternal outcomes of participants receiving either SLMA or ETT while undergoing cesarean section under general anesthesia

Characteristics	SLMA (*n* = 460)	ETT (*n* = 460)	Mean Difference / Odds ratio (95%CI)	*p*-value
Fetal weight (g), mean (SD)	3150.7 (457.9)	3137.8 (417.0)	12.83 (−43.84, 69.49)	0.6570
APGAR score 1 min, median (IQR)	10.0 (9.0, 10.0)	10.0 (9.0, 10.0)	0.00 (0.00, 0.00)	0.9517
APGAR score 5 min, median (IQR)	10.0 (10.0, 10.0)	10.0 (10.0, 10.0)	0.00 (0.00, 0.00)	0.7208
Umbilical venous cord pH, median (IQR)	7.3 (7.3, 7.3)	7.3 (7.3, 7.3)	0.00 (0.00, 0.001)	0.2298
Presence of blood on airway device, n (%)	28 (6.1)	36 (7.8)	0.76 (0.46, 1.27)	0.2999
Incidence of sore throat, n (%)	9 (2.0)	15 (3.3)	0.59 (0.26, 1.37)	0.2146
Incidence of voice hoarseness, n (%)	0 (0.0)	0 (0.0)	–	NA
Patient satisfaction (%), mean (SD)	85.4 (7.6)	84.5 (7.9)	0.86 (−0.14, 1.86)	0.0931
Volume of gastric aspirate (ml), mean (SD)	17.3 (13.4)	17.6 (16.3)	−0.28 (−2.21, 1.65)	0.7756
pH of gastric aspirate, mean (SD)	3.5 (1.4)	3.7 (1.6)	−0.06 (− 0.11, − 0.02)	**0.0309**
pH of SLMA laryngeal surface / ETT cuff, mean (SD)	7.0 (0.2)	7.0 (0.2)	−0.001 (− 0.004, 0.002)	0.5613

Difference between the two categories are expressed as mean difference or median difference and odds ratio with 95% confidence interval for continuous and categorical values, respectively. Categorical values are compared using Chi-Square test and continuous variables are compared using two-sample Student’s t-test or Wilcoxon test. *P*-values of 0.05 or less are considered significant

There was a low incidence of airway complications in this study. Odds of blood on SLMA was lower compared to ETT insertion with OR of 0.76 (95%CI: 0.46, 1.27; *p* = 0.2999). The incidence of sore throat was similar (OR 0.59; 95%CI: 0.26, 1.37, *p* = 0.2146), and there was no voice hoarseness reported in both groups. Patient satisfaction scores were also similar between the groups (MD 0.86; 95%CI: − 0.14, 1.86; *p* = 0.0931).

## Discussion

This large prospective randomized controlled trial involving parturients receiving general anesthesia for cesarean section showed similar first attempt insertion success rates in both SLMA and ETT groups. However, SLMA use was associated with a significant reduction in the time to effective ventilation and less hemodynamic fluctuation in the immediate period after airway insertion. The respiratory, airway, and fetal outcomes were similar between both groups. No evidence of pulmonary aspiration was detected, and the incidence of complications were low in both groups (blood on the airway device, sore throat, voice hoarseness). Maternal satisfaction was also similar.

The first attempt SLMA insertion success rate of 99.1% is comparable to rates previously reported in LMA studies in obstetric general anesthesia (97.7 to 98.0%) [12, 17–19]. The high success rate could be attributed to adherence of the manufacturers’ recommended technique, insertion by experienced anesthetists, and the routine use of SLMA at the study site for cesarean section under general anesthesia. Of note, we achieved a high SLMA insertion success rate despite maintenance of cricoid pressure, which could impede the LMA insertion into the post-cricoid hypopharyngeal space. This issue could have been attenuated by the relative rigidity of the SLMA. The high success rate of SLMA insertion in obstetrics further strengthens our hypothesis that the use of second-generation LMAs such as the SLMA is suitable in selected parturients, and this is consistent with the OAA-DAS recommendations for use in difficult and failed intubation.

Parturients are considered to be at higher risk for gastric regurgitation and pulmonary aspiration. The risk could be exacerbated especially with concomitant obesity, labor pain or opioid analgesia administration. Although this study was not powered to investigate the risk of pulmonary aspiration, we did not detect any clinical evidence of regurgitation or aspiration, including no significant acidification of SLMA laryngeal surface to reflect gastric pH, which is a surrogate indication of the presence of gastric contents in the trachea [20]. The lack of clinical aspiration is possibly due to: (1) the ability of the double-lumen system of second-generation LMAs to facilitate gastric tube insertion and attenuation of intra-gastric fluid volume; (2) a better pharyngeal seal that prevents stomach insufflation and gastric fluid at the hypopharynx from entering the airway; (3) performance of rapid sequence induction and cricoid pressure; (4) careful patient selection with exclusion of parturients at risk of gastric regurgitation; and (5) adherence to the minimum recommended fasting time**.** We also requested the obstetricians to reduce fundal pressure for all cases, though the benefit of this in terms of aspiration risk is uncertain.

Our results concurred with previous studies evaluating the role of supraglottic airway devices in parturients receiving general anesthesia for cesarean section. The prospective study using the Classic™ LMA in 1067 parturients undergoing elective cesarean section by Han et al. in 2001 detected no aspiration or regurgitation [19]. The use of the ProSeal LMA in 3000 parturients by Halaseh et al. reported a single event of gastric regurgitation after LMA insertion, but with no clinical evidence of aspiration [17]. More recently, our team demonstrated the safety of SLMA use for airway management in obstetric general anesthesia, in a cohort study involving 700 low-risk parturients undergoing elective and urgent cesarean section [12], followed by a higher-risk 584 women requiring emergent Category 2 or 3 cesarean section, of which 38.4% were in active labor [18]. Both studies reported a high first attempt success rate of SLMA insertion of 98% and no clinical evidence of regurgitation or aspiration.

There are several limitations in our study. Our participants were carefully selected to reduce the risk of gastric regurgitation and our study was not powered to detect such rare events. By extension, we are also unable to extrapolate our findings to parturients who are at a higher risk of pulmonary aspiration. Based on a previous study by Halaseh et al., with a baseline risk of aspiration of 1:1000, at least 3000 cases are required based on a cohort study [17]. This would make the study feasibility difficult given the large numbers. The cesarean section rate is 35% in our institution and the SLMA technique for general anesthesia is used in 2000 deliveries annually. Since we have adopted the SLMA in airway management of selected parturients, we have not noted any adverse events (aspiration). However, the current practice of endotracheal intubation for cesarean section should still be advocated as pulmonary aspiration is one of the main concerns with SLMA use in obstetrics, for which our study was not powered to detect. We noted that the familiarity with the use of SLMA in general anesthesia for cesarean section and less familiarity with endotracheal intubation could have led to faster time to ventilation and high insertion success rate with SLMA in this study center. Thus, these findings may not be applicable to other centers. Also, cricoid pressure was applied by the anesthetic nurses, who were briefed on the study and the need for consistency. They applied cricoid pressure according to routine hospital practice, but cricoid pressure was not directly measured. Although the obstetricians were not blinded, they were instructed to reduce fundal pressure uniformly for all cases. Lastly, there is no reliable method to blind the anesthesiologists who performed the airway management in this study. Hence, outcome measures including the primary outcome could be influenced by the experience and familiarity with the airway devices used.

The faster speed to establish a secure airway and the reduced risk of hemodynamic fluctuations renders potential justification for future studies on the effectiveness of second-generation LMAs in the obstetric population especially in difficult obstetric airway management. Higher-risk obstetric parturients with medical conditions such as preeclampsia and cardiac disease could benefit from reduced hemodynamic alterations associated with SLMA use [21, 22]. Furthermore, during emergency airway management, a shorter time to ventilation could reduce potential hypoxia [8, 23].

## Conclusion

We found that in parturients undergoing elective cesarean section under general anesthesia, the SLMA group has similar high first attempt success rate, while potentially reducing the time taken to achieve effective ventilation and less hemodynamic changes, as compared to ETT group. However, there is still limited information of the safety of SLMA use, especially pertaining to pulmonary aspiration risk. Given the higher incidence of difficult and failed intubation in obstetrics, our findings support the current DAS-OAA guidelines in recommending the use of second-generation LMAs as a second-line airway device, in place of persistent attempts at endotracheal intubation.

## Data Availability

The datasets generated and analyzed for this manuscript are not publicly available, but could be obtained from the corresponding author on reasonable request.

## Abbreviations

ASA: American Society of Anesthesiology
BMI: Body mass index
CI: Confidence interval
DAS: Difficult Airway Society
ETT: Endotracheal intubation
IQR: Interquartile range
LMA: Laryngeal mask airway
MD: Mean difference
OAA: Obstetric Anaesthetists’ Association
OR: Odds ratio
RSI: Rapid sequence induction
SAD: Supraglottic airway devices
SD: Standard deviation
SLMA: Supreme laryngeal mask airway
